# Selective evolution of methanogenic communities by β-lactam antibiotics promotes methane production

**DOI:** 10.1128/spectrum.01693-24

**Published:** 2025-04-22

**Authors:** Yuting Zhe, Huaigang Cheng, Danjing Ding, Yueyue He, Zhuohui Ma, Jing Shen

**Affiliations:** 1 Institute of Resources and Environmental Engineering, Engineering Research Center of CO2 Emission Reduction and Resource Utilization-Ministry of Education of the People's Republic of China, Shanxi University 12441, Taiyuan, Shanxi, China; 2 Salt Lake Chemical Industry Large Series Research Facility, Qinghai University 207475, Xining, Qinghai, China; University of Porto, Porto, Portugal

**Keywords:** biomethane, methanogens, selective evolution of communities, β-lactam antibiotics, ARGs

## Abstract

Biomethane, a clean energy source, plays a crucial role in addressing global energy and environmental challenges. However, its production has long been limited by the complexity of methanogenic precursor synthesis and the involvement of diverse microbial strains, creating a bottleneck in methane conversion. To address this, the present study explores the potential of the β-lactam antibiotic amoxicillin (AMX) to enhance methanogenesis by selectively shaping microbial communities while preserving core methanogenic pathways. Results indicate that the addition of AMX significantly improves methanogenic performance. By day 25, methane yield increased by 3.89-fold, and carbon substrate utilization efficiency improved by 15.5% compared to the control group. This enhancement is linked to the selective effect of AMX on the methanogenic community, leading to a 61.51% increase in the relative abundance of methanogenic archaea. This approach optimizes the process by bypassing three stages of methane precursor synthesis, reducing interfering bacteria, and improving methanogenic efficiency and process controllability, all while showing no significant negative environmental impacts from the added antibiotics.

**IMPORTANCE:**

This study holds significant importance in advancing biomethane production, a clean and renewable energy source. By utilizing amoxicillin, the research promotes the selective growth of methane-producing microbes while suppressing harmful ones, resulting in faster and more efficient methane production with enhanced process control. In addition, amoxicillin degrades rapidly within the system, resulting in minimal negative environmental impact. This work provides a promising and eco-friendly approach to optimize biomethane production, offering a viable and sustainable solution for energy generation from waste.

## INTRODUCTION

Biomethane (1), produced by methanogens through microbial activity under anaerobic conditions, has been studied extensively since the early 20th century. In the energy sector, biomethane is recognized as a clean energy alternative (2), and it plays a crucial role in regulating greenhouse gas balance on Earth in the environmental and ecological domains (3). The low efficiency (0.25–0.6 m³ CH₄/m³/day) and limited controllability of microbial methanogenesis are significant barriers to its industrial application. Addressing these issues is vital for the industrialization of microbial methanogenesis technology.

Microbial methanogenesis is a complex cooperative process involving diverse microbial communities, such as depolymerizing bacteria, fermentative bacteria, hydrogen and acetic acid-producing bacteria, and methanogens. These microorganisms participate in four stages of anaerobic digestion (AD): hydrolysis, acidogenesis, hydrogen and acetic acid production, and methanogenesis (4). Methanogens, as the final participants, convert acetate, CO₂, and H₂ into methane. The synthesis of methanogenic precursors relies on the first three stages, resulting in a slow growth of methanogens (5). The numerous steps and involvement of various microbial strains in the synthesis of methanogenic precursors result in low gas production efficiency and uncontrollable methanogenesis processes (6, 7).

Simplifying the anaerobic digestion (AD) methanogenesis process can enhance the controllability of biological reactions and improve the predictability of their outcomes. Methanogens are capable of directly utilizing substrates such as sodium acetate and CO₂ (8, 9), simplifying methane production. Under these conditions, other microorganisms, except methanogens and hydrogenogens, become less functional or even act as interfering bacteria (10) because they may compete with methanogens for nutrients (11) or produce harmful by-products that inhibit methane production (12). However, merely changing substrates to prioritize the methanogenesis stage does not prevent the normal activity of preceding stages, continuing to limit the growth of methanogens. Achieving selective evolution of microbial communities to preserve the core methanogenesis stage is the key scientific problem addressed in this study.

Antibiotics are widely used to reduce interfering bacteria in medical (13), livestock (14), and food industries (15). Several studies have demonstrated the potential of antibiotics to achieve selective evolution of microbial communities: in medicine, clarithromycin and amoxicillin selectively inhibit and eliminate *Helicobacter pylori* with minimal impact on beneficial gut bacteria such as lactobacilli and bifidobacteria (16); in cheese fermentation, natamycin selectively inhibits mold and yeast growth without affecting lactic acid bacteria fermentation (17); in isolating lactobacilli from fecal samples, vancomycin-containing media selectively inhibit most Gram-positive bacteria, allowing selective isolation of lactobacilli (18). Choosing an antibiotic that achieves selective evolution of microbial communities while retaining the core methanogenesis stage is crucial. β-Lactam antibiotics have a unique antibacterial mechanism that interferes with bacterial cell wall synthesis (19), which does not affect methanogens as they lack cell walls. Theoretically, β-lactam antibiotics can selectively inhibit non-methanogenic microorganisms in mixed microbial communities (20), thereby indirectly promoting methane production by reducing competition. This approach, which inhibits other microbial strains while retaining the core methanogenesis stage, provides a new perspective on understanding and utilizing antibiotics in the methanogenesis process and their impact on the dynamic relationships within microbial communities.

This study aims to enhance methanogenesis efficiency and process controllability by selectively inhibiting non-methanogenic bacteria with β-lactam antibiotics, thereby reducing interfering bacteria and optimizing the elimination of three methanogenic precursor synthesis stages. In addition, this study assesses the environmental safety of these antibiotics to ensure a balance between sustainable energy production and environmental protection.

## MATERIALS AND METHODS

### Construction of methanogenic microbial reactors

The anaerobic sludge used in this study was sourced from the anaerobic fermentation pool of the Shanxi Yuanping Muyuan Agriculture and Animal Limited Company, located in Xinzhou City, Shanxi Province, China. The properties of the anaerobic sludge are shown in Table 1. Methanogens were cultured in a specialized medium with the following composition: K₂HPO₄ 0.4 g/L, KH₂PO₄ 0.4 g/L, MgCl₂ 1.0 g/L, NH₄Cl 1.0 g/L, 0.1% resazurin 2.0 mL/L, cysteine 0.5 g/L, Na₂S 0.5 g/L, NaHCO₃ 3.0 g/L, CH₃COONa 5.0 g/L, trace element solution 1.0 mL/L, and vitamin solution 1.0 mL/L.

**TABLE 1:** The properties of the anaerobic sludge

Parameters	Anaerobic sludge
pH	7.5
TS^*a*^ (mg/L)	130
VS^*b*^ (mg/L)	70
TCOD^*c*^ (mg/L)	4,100
SCOD^*d*^ (mg/L)	2,680
TP^*e*^ (mg/L)	138
TN^*f*^ (mg/L)	2,100
NH4^+^-N^*g*^ (mg/L)	940

^*a*^ Total solid.

^*b*^ Volatile solid.

^*c*^ Total chemical oxygen demand.

^*d*^ Soluble chemical oxygen demand.

^*e*^ Total phosphorus.

^*f*^ Total nitrogen.

^*g*^ Ammonium nitrogen.

Methanogenic microbial reactors were constructed using 500 mL PET bottles equipped with dual-port caps and 10 mL syringes. Methanogens were utilized to directly convert sodium acetate into methane, which was captured in the syringes. The medium was deoxygenated by heating and allowed to cool to room temperature prior to inoculation. A 1:1 vol ratio of medium to anaerobic sludge was mixed and added to the reactors, which were then purged with nitrogen gas for 30 minutes to establish an anaerobic environment. The reactors were maintained at a constant temperature of 37 ± 1°C, and the reactor solution was left unchanged throughout the experiment.

### Experimental groups and design

In this study, amoxicillin (AMX), a typical β-lactam antibiotic, was used as the experimental group. In addition, a tetracycline group (TET) and a blank control group (CG) without antibiotics were established for comparison. The effects of these two antibiotics on the methanogenesis process were investigated at a concentration of 1 × 10⁵ u/L. For AMX, TET, and CG, 1 × 10⁵ u/L corresponds to 100 mg/L, 100 mg/L, and 60 mg/L, respectively. Three parallel reactors were set up simultaneously, and the experiments were repeated three times. Error bars represent the standard deviation of data from the three repeated experiments, ensuring the reliability of the results. One-way analysis of variance (ANOVA) was used to evaluate the significance of differences among experimental groups. Results were considered statistically significant when *P* < 0.05 and highly significant when *P* < 0.01.

### Sample collection and analysis

Methane production was measured every 24 hours using syringes. Reactor solution samples were collected every 5 days for analysis, with the total experiment duration set at 25 days. Methane content in the gas samples was determined using a gas chromatograph (SP-7890Plus, Lunan Rui Hong, Tengzhou, China). Sodium acetate concentrations were measured with a high-performance liquid chromatograph (HPLC) (1260 Infinity II, Agilent, USA) equipped with a 120 EC-C18 column (Agilent, USA). Detection method parameters: Mobile phase (0.025M sulfuric acid), detection wavelength: 210 nm, flow rate: 0.8 mL/min, column temperature: 30°C, injection volume: 20 µL. The morphology of methanogens was observed using a scanning electron microscope (TM3030, Hitachi, Japan). Soluble protein (SP) and soluble sugar (SS) concentrations were measured using the Coomassie Brilliant Blue method and the anthrone colorimetric method, respectively. AMX concentrations were determined by high-performance liquid chromatography (HPLC) (1260 Infinity II, Agilent, USA) with an EC-C18 column (Agilent, USA). Microbial community dynamics were analyzed every 5 days using 16S rRNA sequencing with the Illumina Miseq platform. Antibiotic resistance genes (ARGs) were detected through metagenomic sequencing.

### Model construction methods

The model is constructed using cellular automata (CA) and integrates community evolution with the Michaelis-Menten kinetics model. The community evolution component consists of three sub-models: the Monod, Logistic, and Lotka-Volterra models. The spatial distribution map of the community is represented by a grid with a cell size of 20,000 × 20,000, where each cell corresponds to the space occupied by a single bacterium. This section does not include a quantifiable scale. In addition, these data are simulations and do not reflect the actual distribution of the different types of microorganisms.

The Monod model describes the growth rate of microorganisms as a function of substrate concentrations:


$$\mu_{i}=\mu_{max,i}\frac{S}{K_{s,i}+S}$$

where *μ_i_* represents the specific growth rate of the *i*th microorganism, *μ_max,I_* is the maximum specific growth rate of the *i*th microorganism, *S* is the substrate concentration, and *K_s,i_* is the half-saturation constant of the *i*th microorganism, representing the substrate concentration at which the growth rate is half of its maximum value.

The Logistic model is used to describe the growth of a single population under limited resources:


$$\frac{{dN}_{i}}{dt}=\mu_{i}N_{i}(1-\frac{N_{i}}{K_{i}})$$

where $\frac{{dN}_{i}}{dt}$ is the growth rate of the *i*th population, *N_i_* is the number of the *i*th population, and *K_i_* is the carrying capacity of the *i*th population.

The Lotka-Volterra model is used to describe the competitive relationships between multiple populations:


$$\frac{{dN}_{i}}{dt}=\mu_{i}N_{i}(1-\frac{\sum_{j}\alpha_{ij}N_{j}}{K_{i}})$$

where *α_ij_* is the competition coefficient representing the impact of the *j*th population on the *i*th population, and *∑_j_α_ij_N_j_* represents the total competitive pressure from all competing populations on the *i*th population.

By combining the above three models, we obtain the following modified community evolution model that considers the effects of substrate concentration, population density, and interspecies competition on microbial growth:


$$\frac{{dN}_{i}}{dt}=\mu_{max,i}\frac{S}{K_{s,i}+S}N_{i}(1-\frac{\sum_{j}\alpha_{ij}N_{j}}{K_{i}})$$

The Michaelis-Menten kinetics model is used to describe the relationship between the methanogenic reaction rate and substrate concentration:


$$V=\frac{V_{max}∙[S]}{K_{m}+[S]}$$

where *V* is the reaction rate, *V_max_* is the maximum reaction rate, [*S*] is the substrate concentration, and *K_m_* is the Michaelis constant, representing the substrate concentration at which the reaction rate is half of *V_max_*.

## RESULTS AND DISCUSSION

### Behavior and morphological changes of methanogenesis

**Fig 1:**
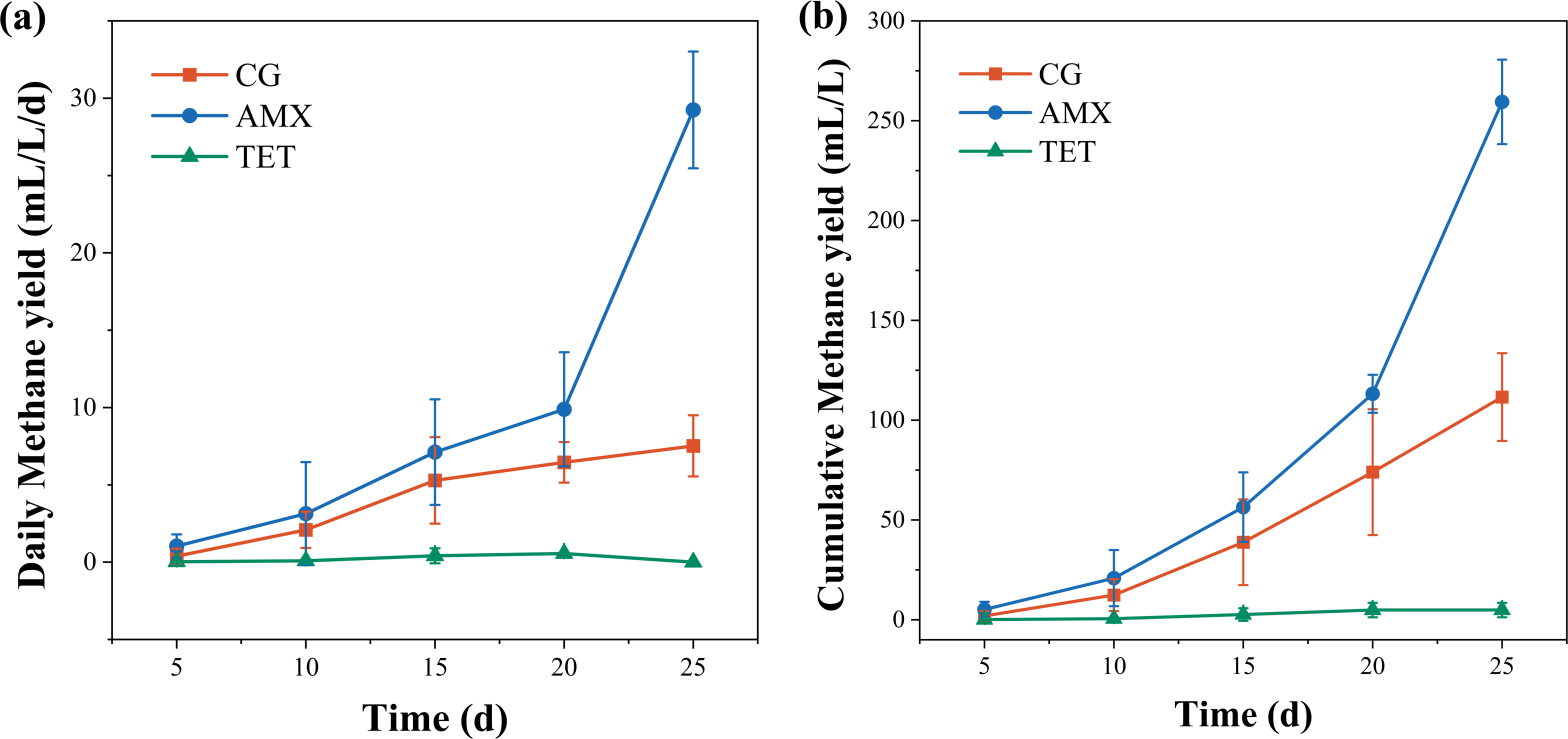
Trends in methanogenic performance: (a) daily methane yield (b) cumulative methane yield.

The behavior of methanogenesis reflects the direct outcome of the microbial methanogenic process. Figure 1 illustrates the trends in methanogenic performance over 25 days. As shown in Fig. 1a, in the absence of antibiotics, the daily methane yield exhibits a slow increasing trend. In the TET group, daily methane yield significantly decreases compared to the control group and ceases entirely by day 25, indicating that TET inhibits the methanogenesis process. By contrast, the AMX group shows a higher daily methane yield than the control group, particularly after day 20, with methanogenic performance rapidly increasing and reaching 29.2 mL/L·d by day 25, which is 3.89 times higher than that of the control group. Figure 1b depicts the cumulative methane yield, following the trend AMX > CG > TET. These results demonstrate that β-lactam antibiotics promote methane production, whereas TET inhibits it.

**Fig 2:**
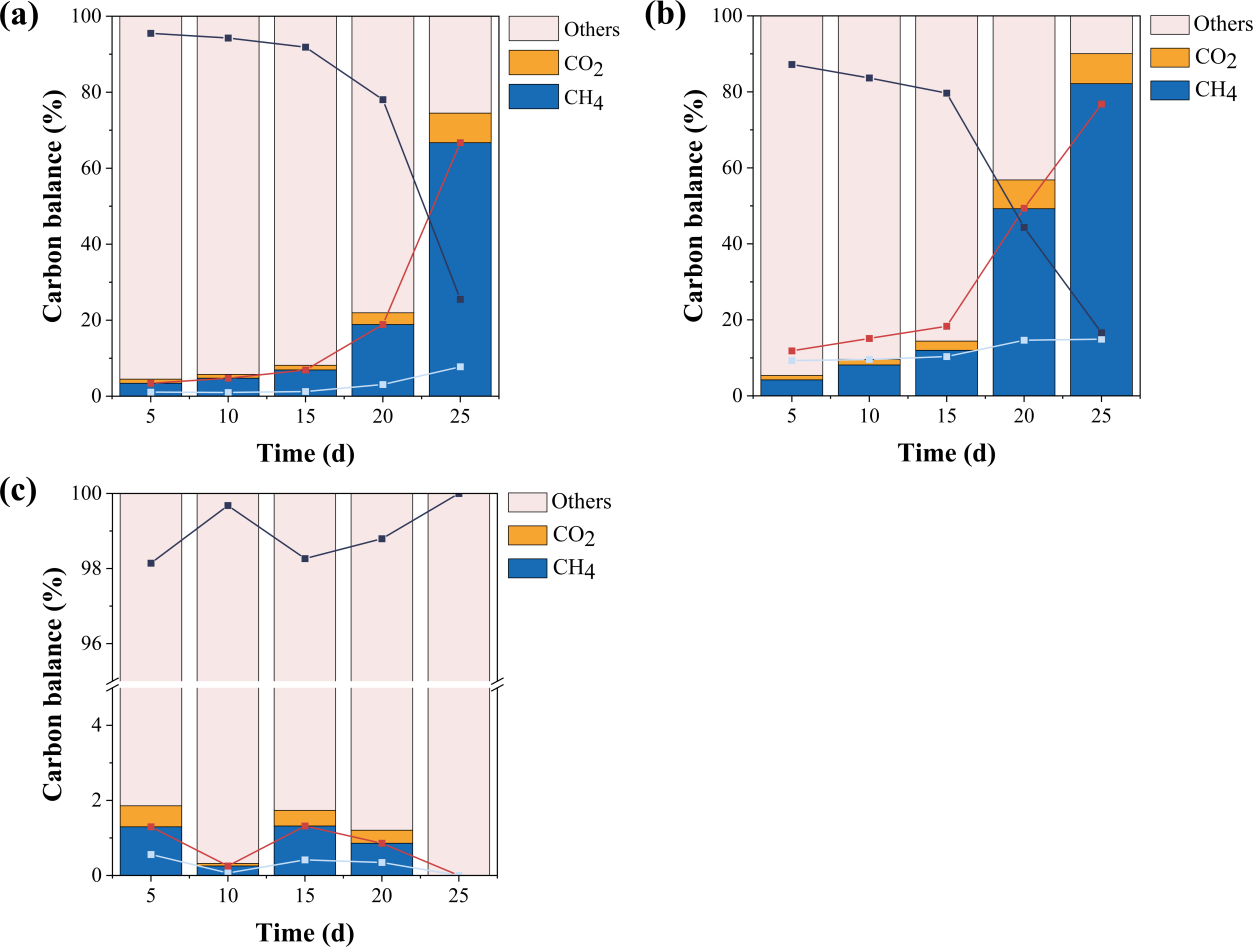
Carbon balance of methanogenic processes: (a) CG (b) AMX, and (c) TET.

Carbon balance reflects the utilization of carbon elements from substrates during the microbial methanogenesis process. To examine the effect of β-lactam antibiotics on the carbon utilization rate, trends in carbon balance are presented in Fig. 2. Figure 2a depicts the changes in carbon balance in the control group. On the 5th day, only 3.4% of the carbon elements from the substrates were converted into methane. This gradually increased, reaching 66.7% conversion to methane and 7.8% to CO₂ by the 25th day. Figure 2b shows the trend of carbon balance in the AMX group. On the 5th day, 4.2% of the substrate carbon elements were converted into methane, and by the 25th day, the conversion rate reached 82.2%. This suggests that β-lactam antibiotics like AMX can increase the conversion rate of substrate carbon elements to methane, promoting the methanogenesis process. In Fig. 2C, where the methane conversion rate decreased from 1.3% on the 5th day to 0% on the 25th day, indicating that TET inhibits the methanogenesis process and prevents the utilization of carbon elements for methane production.

**Fig 3:**
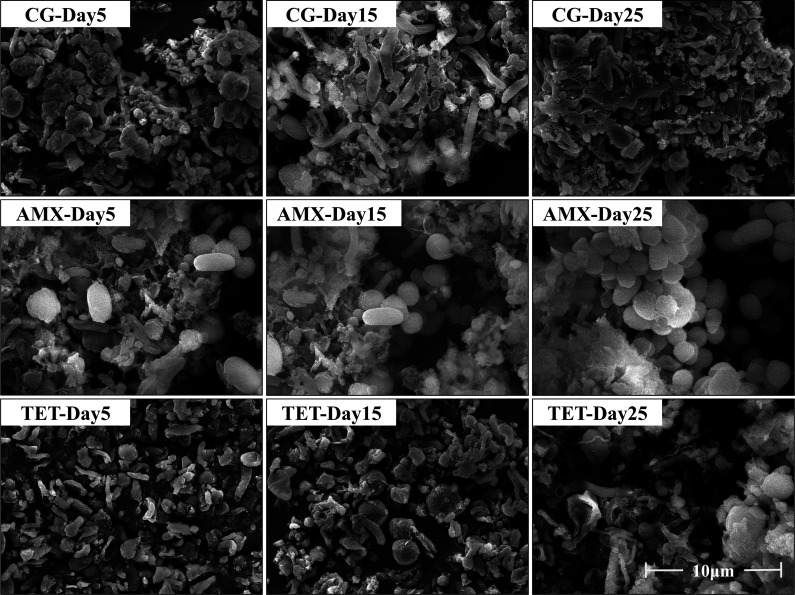
Morphology of methanogenic communities on the 5th, 15th, and 25th days of cultivation at 20,000× magnification.

The micro-morphology of methanogenic communities reflects the microbial response to antibiotic stress; however, the images do not clearly distinguish methanogens from other bacteria. The observed morphological changes are presented in Fig. 3. On day 5, cells in the control group appeared intact; in the AMX group, some cells remained intact, while others ruptured and leaked cytoplasm; and in the TET group, most cells displayed deformation or lysis. By day 15, cell numbers in the control group slightly increased without notable morphological changes; in the AMX group, some cells remained intact and increased in number, while others showed rupture; and in the TET group, no significant changes were observed. By day 25, the control group maintained microbial diversity, the AMX group exhibited a significant increase in cell numbers, and the TET group demonstrated extensive cell lysis.

It should be noted that the observed morphological changes do not clearly distinguish methanogens from other microorganisms. Based on existing literature and previous findings, it is hypothesized that AMX strongly inhibits certain interfering bacteria while exerting a relatively minor impact on methanogens, thereby providing methanogens with a competitive advantage. By contrast, TET likely exerts broad inhibitory effects on both methanogens and interfering bacteria. However, these hypotheses require further validation through molecular analyses and functional studies.

In summary, β-lactam antibiotics, exemplified by AMX, can enhance the methanogenesis process, as demonstrated by a 3.98-fold increase in methane yield and a 15.5% conversion rate of substrate carbon elements to methane on the 25th day. Morphological observations suggest that AMX may selectively inhibit certain microorganisms, thereby reducing competition and creating favorable conditions for methanogenesis. Although further studies employing molecular and functional analyses are needed to provide a more comprehensive understanding of microbial community changes, these preliminary findings indicate that such changes may play a pivotal role in AMX-enhanced methanogenesis, which will be further investigated in subsequent research.

### Effect of antibiotics on the evolution of methanogenic communities

This section investigates changes in microbial biomass and community evolution to identify the key factors through which β-lactam antibiotics influence the methanogenic process—whether these effects arise from alterations in microbial biomass or community composition.

**Fig 4:**
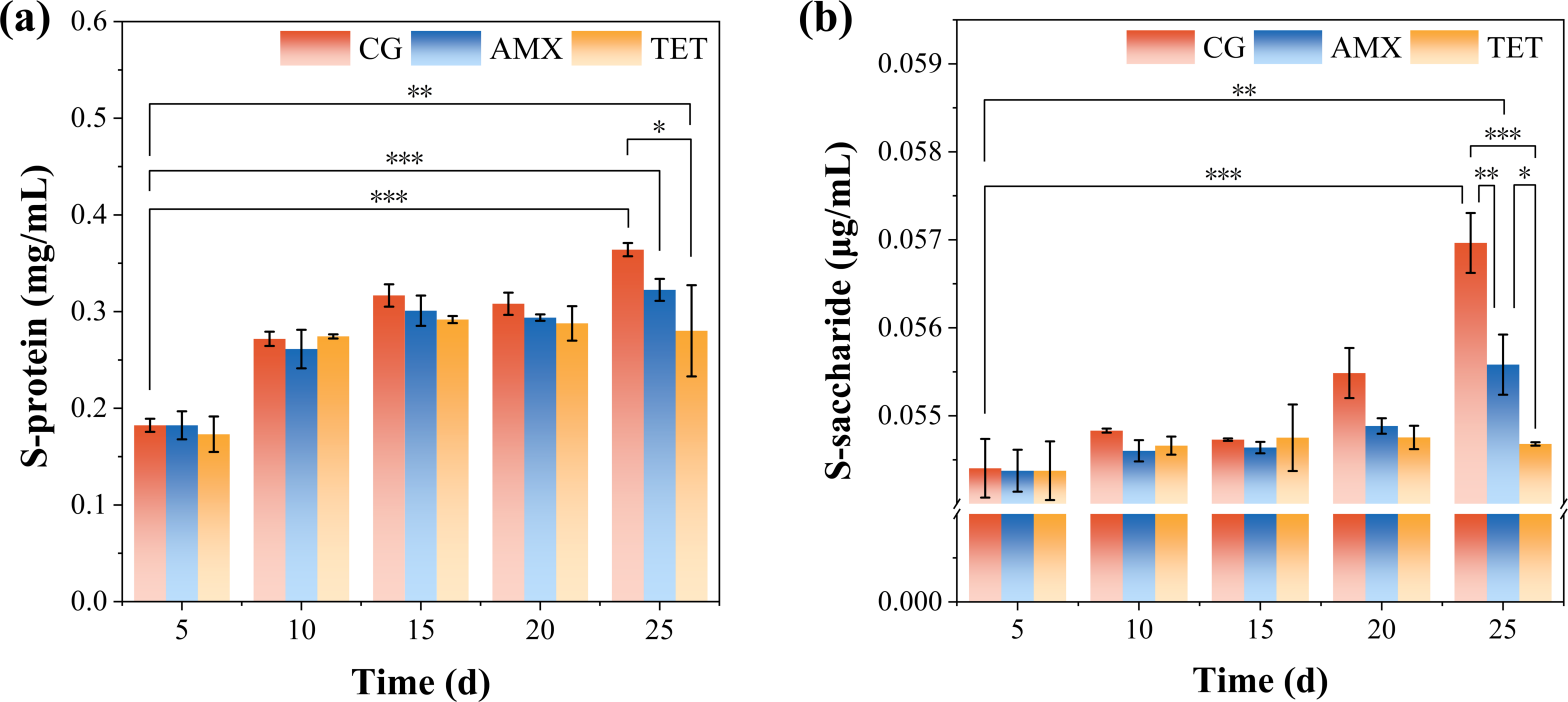
Changes in the content of (a) soluble protein and (b) soluble sugars in methanogenic communities.

Changes in microbial biomass were assessed using soluble protein and soluble sugar contents as indicators. Soluble protein and soluble sugars serve as energy sources for cells and important growth regulators, reflecting variations in microbial biomass. The trend of soluble protein changes is illustrated in Fig. 4a. Both the control group and the AMX group exhibited an overall increasing trend over time. On the 5th day of inoculation, soluble protein content in the AMX group was not significantly different from that in the control group; however, as cultivation progressed, it gradually decreased below the levels observed in the control group. This reduction may be attributed to soluble protein content representing the entire system, with AMX inhibiting the growth and reproduction of certain interfering bacteria, thereby leading to lower soluble protein levels in the AMX group. By contrast, the TET group showed a trend of initial increase followed by a decrease, likely due to TET-inducing microbial cell death and system instability.

The changes in soluble sugars (Fig. 4b) followed a trend similar to that of soluble protein, with the AMX group and the control group increasing slowly during the first 15 days and then more rapidly thereafter. The final observed trend was CG > AMX > TET. However, the differences in soluble sugar content among the groups, particularly between CG and AMX, were relatively small (less than 5%). Although statistically significant, these differences may have limited practical significance.

These findings suggest that β-lactam antibiotics, such as AMX, reduce microbial biomass, as indicated by lower soluble protein and soluble sugar contents. In addition, the data imply that microbial biomass may not be the primary factor driving methanogenesis under β-lactam antibiotic treatment.

**Fig 5:**
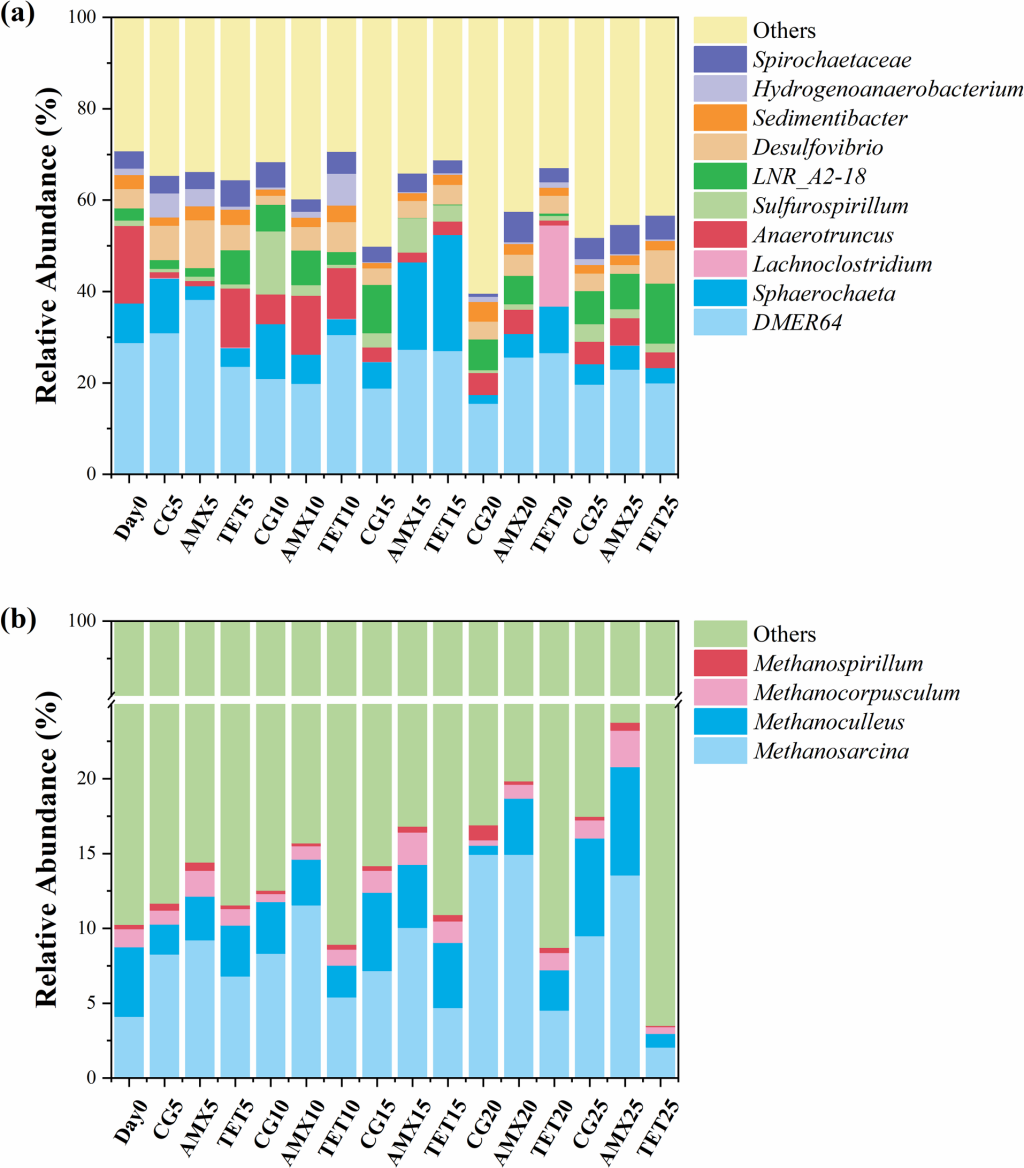
Evolution of the methanogenic community composition: (a) bacterial community evolution (b) archaeal community evolution.

The microbial community evolution process reflects the patterns of community changes over time. Figure 5a and b illustrates the relative abundance changes of bacteria and archaea at the genus level, respectively. In Fig. 5a, the abundances of *Sulfurospirillum* and *Desulfovibrio* decreased by 50.6% and 49.6%, respectively, compared to the control group, indicating that AMX inhibited these bacteria. The primary hydrogen-producing bacterium, *Hydrogenoanaerobacterium* (21) was significantly inhibited by AMX. By day 20, the abundance of *Hydrogenoanaerobacterium* in the AMX group had decreased by 67.1% compared to the control group, and by the 25th day, it decreased by 78.3%. Although the reduction of hydrogen-producing bacteria does not affect the methanogenesis process using sodium acetate as a substrate, it negatively impacts the process using CO₂ as a substrate, as CO₂ reduction to methane relies on H₂ as a reducing equivalent. The absence of hydrogenogens may necessitate the use of exogenous H₂ or other reducing equivalents to ensure methane production. It should be noted that the hypothesized impact of hydrogen production inhibition on hydrogenotrophic methanogenesis remains speculative, as hydrogenotrophic methanogenesis activity was not separately measured.

As shown in Fig. 5b, the main methanogens in the system are *Methanosarcina*, *Methanoculleus*, *Methanocorpusculum*, and *Methanospirillum*. In the control group, the relative abundance of methanogens exhibited a slow increasing trend, rising from 10.26% before inoculation to 17.45% on the 25th day. In the AMX group, methanogens displayed a more rapid growth trend, increasing from 10.26% to 23.76%, with a growth rate 61.51% higher than the control group. This suggests that AMX does not inhibit methanogens; rather, as the number of interfering bacteria decreases, it makes energy and nutrients in the reaction system more accessible to methanogens, expanding their living space and promoting their growth and reproduction. By contrast, TET exhibited a strong inhibitory effect on methanogens, reducing their relative abundance from 10.26% to 3.51%.

### Mechanism and model analysis of AMX-promoted methanogenesis process

Adding β-lactam antibiotics such as AMX to inhibit interfering bacteria that might interfere with methane production represents a novel strategy to simplify the methanogenesis process. At the macroscopic level, β-lactam antibiotics indirectly promote the growth of methanogens by selectively inhibiting interfering bacteria (*Sulfurospirillum* and *Desulfovibrio* abundances decreased by 50.6% and 49.6%, respectively, compared to the control group). This selective evolution of the community accelerates methane production. The inhibition of interfering bacteria by AMX enables more energy and nutrients in the reaction system to be allocated to methanogens, thereby expanding their ecological niche and promoting their growth and reproduction.

Moreover, low doses of β-lactam antibiotics might directly stimulate methanogens, thereby enhancing methane production. In anaerobic digestion, studies have demonstrated that low doses of tar (22), low-dose ozone pretreatment (23), and low-intensity ultrasound (24) can exhibit environmental toxic excitation effects. Similarly, low-dose antibiotics have been shown to exert stimulatory effects in different biochemical environments, such as promoting plant growth (25) and accelerating bacterial growth (26). It can be inferred that low doses of β-lactam antibiotics may excite methanogens and further increase methane production.

On a microscopic level, the principal mechanism of action is by inhibiting the DAla-DAla transpeptidase and carboxypeptidase activity of the PBPs required for peptidoglycan biosynthesis (27). The lack of sensitivity of archaea to beta-lactams arises from the fact that they do not have peptidoglycan and hence do not require the activity of PBPs. Therefore, AMX can selectively inhibit specific types of bacteria without affecting methanogens. For example, introducing penicillins and cephalosporins into agar media can significantly inhibit strains such as *Staphylococcus aureus*, *Escherichia coli*, and *Klebsiella pneumoniae*, preventing contamination by these bacteria (28). In this study, AMX selectively inhibited *Sulfurospirillum* (inhibition rate 50.6%) and *Desulfovibrio* (inhibition rate 49.6%), facilitating the growth of methanogens.

However, AMX also exhibits the side effect of inhibiting hydrogenogens (reduced by 78.3%). When CO₂ is used as a substrate, the lack of hydrogen produced by hydrogenogens to reduce CO₂ to methane necessitates the addition of exogenous hydrogen or other reducing equivalents to sustain methane production (29–31).

By combining this specific substrate utilization and inhibition strategy, the AD process can be optimized to some extent, accelerating the enrichment of methanogens and enhancing methane yield. This method helps simplify complex microbial communities, making the biological reaction process more controllable and its outcomes more predictable. In addition, this strategy of inhibiting interfering bacteria offers environmentally friendly benefits, making it a promising tool for industries related to microbial fermentation and microbial catalytic conversion.

**Fig 6:**
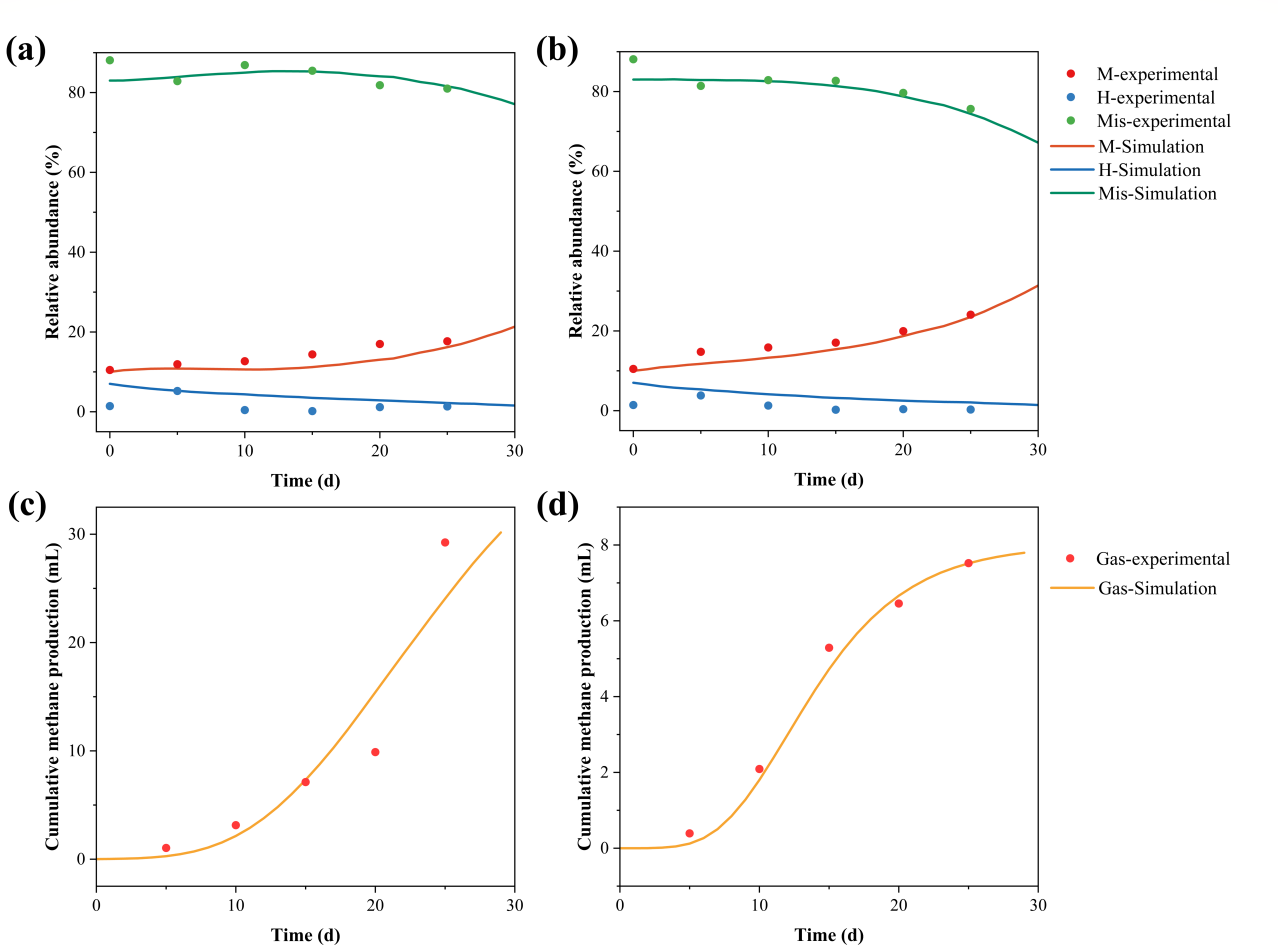
Data fitting of the methanogenic community evolution model: (a) CG community evolution, (b) AMX community evolution, (c) CG methane production, and (d) AMX methane production.

**Fig 7:**
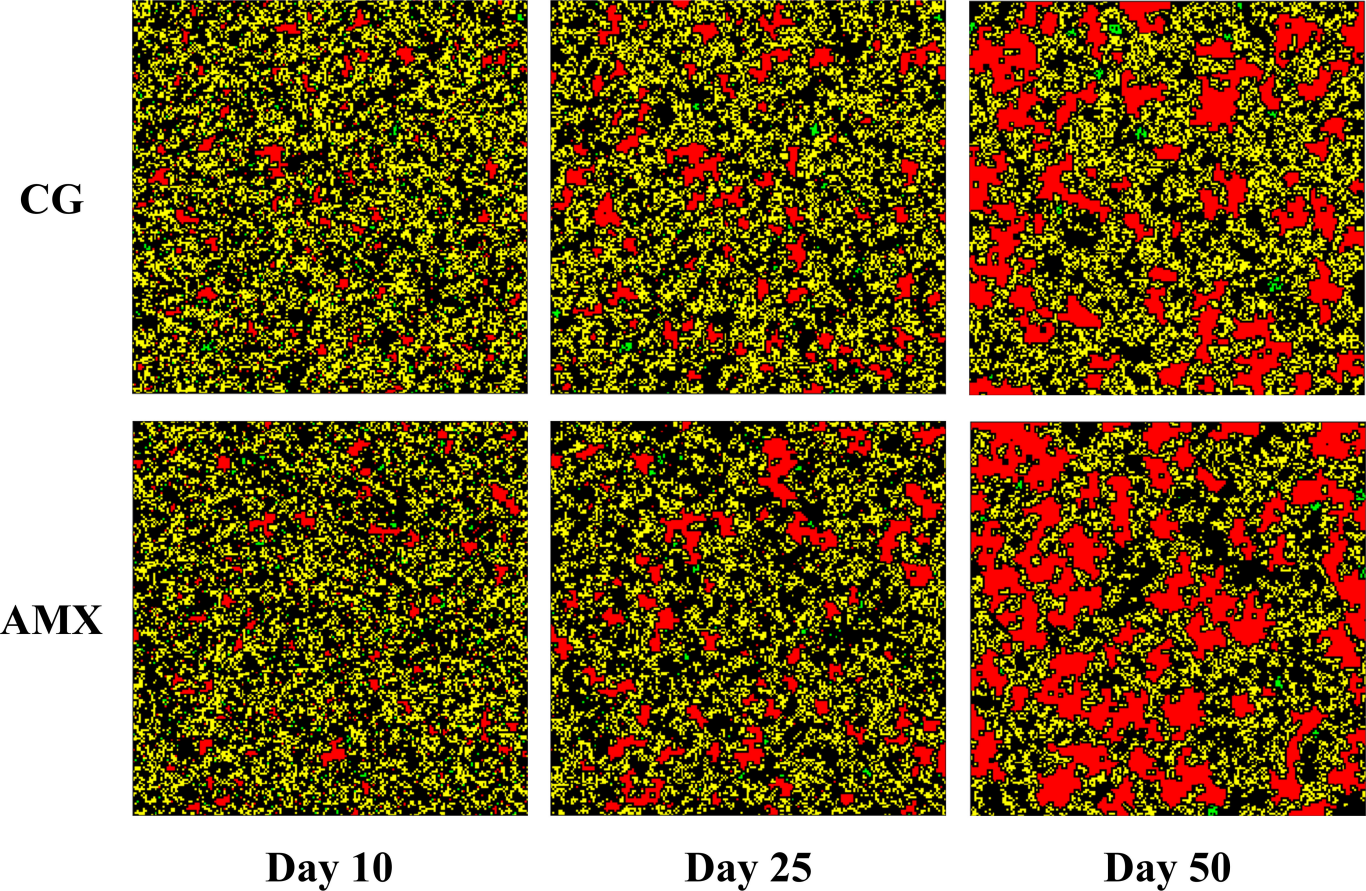
Community spatial distribution simulation (red: methanogens, yellow: interfering bacteria, green: hydrogenogens).

To better elucidate the mechanism by which AMX promotes the methanogenesis process, this section presents a cellular automaton (CA) model to simulate the interspecific competition and spatial distribution changes within microbial communities under the influence of AMX. The developed model was fitted to experimental data to validate its reliability, and the fitting results are shown in Fig. 6. The model’s evaluation parameters are summarized in Table 2. MAE (Mean Absolute Error), MSE (Mean Squared Error), and RMSE (Root Mean Squared Error) are statistical metrics used to evaluate model performance, with lower values indicating better predictions. In Table 2, the low error values for methanogens, hydrogenogens, and interfering bacteria in both the CG and AMX groups suggest the model effectively captures community dynamics. The results indicate that the model demonstrates a strong fitting performance in capturing the evolution of the microbial community. The lower fitting accuracy of methane yield is because it is attributed to its dependence not only on the number of methanogens but also on the activity of methanogens, substrate concentration, gas collection errors, and other factors, and other variables, leading to limited fitting accuracy. As shown in Fig. 6, AMX significantly increased the relative abundance of methanogens while reducing the relative abundance of interfering bacteria, with community changes positively correlated with the methanogenesis rate. The spatial distribution changes illustrated in Fig. 7 visually depict the community evolution process and predict the outcomes after 50 days of cultivation. Methanogens gradually transition from a relatively dispersed state to occupying a dominant position, whereas hydrogenogens become sparsely distributed. As cultivation time increases, the distribution area of the AMX group becomes significantly larger than that of the control group, providing supporting evidence that AMX significantly promotes the growth of methanogens while inhibiting interfering bacteria.

**TABLE 2:** Evaluation of model performance

Group	Community	MAE	MSE	RMSE
CG	Methanogens	0.0203	0.0006	0.0236
	Hydrogenogens	0.0258	0.0010	0.0320
	Miscellaneous	0.0183	0.0006	0.0245
	Methane production	0.2660	0.1028	0.3206
AMX	Methanogens	0.0158	0.0003	0.0185
	Hydrogenogens	0.0281	0.0010	0.0311
	Miscellaneous	0.0172	0.0005	0.0233
	Methane production	2.5352	11.8095	3.4365

### Degradation of antibiotics and changes in antibiotic resistance genes during methanogenesis

This section evaluates whether the addition of antibiotics adversely impacts the environment by introducing more antibiotic resistance genes (ARGs). Previous studies have demonstrated that antibiotics are often partially degraded during anaerobic digestion (32, 33), accompanied by a reduction in the abundance of resistance genes (34, 35). To assess whether similar effects occur in the reactors used in this study, the degradation of AMX and changes in the distribution and abundance of ARGs are analyzed and compared.

**Fig 8:**
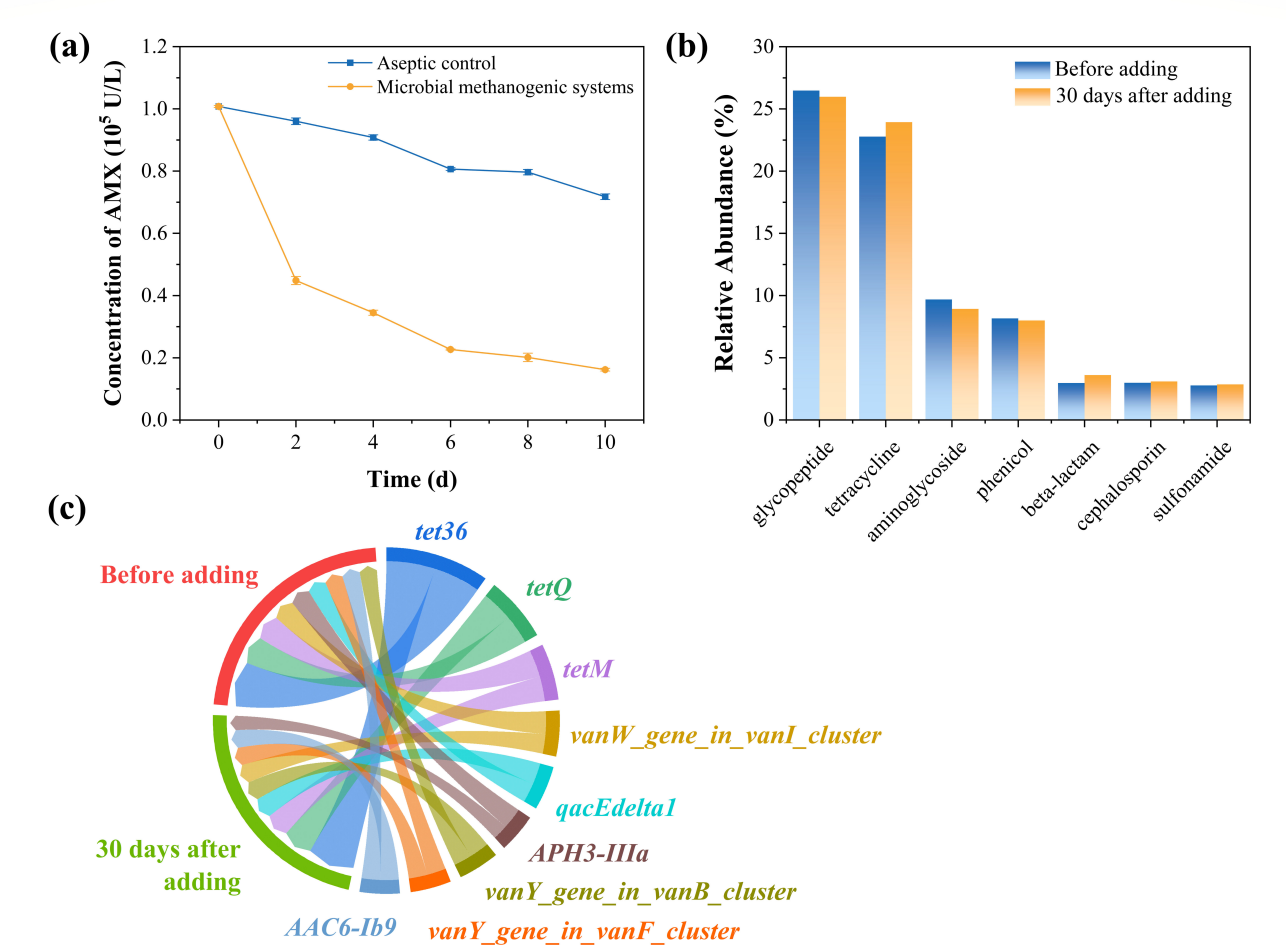
Environmental impact before and after the addition of β-lactam antibiotics: (**a**) degradation curve of AMX, (**b**) circos plot of ARGs distribution, and (**C**) relative abundance bar chart of ARGs.

To evaluate the degradation of antibiotics in the reactors, the AMX content was measured over a 10-day period, as shown in Fig. 8a. Compared to the sterile control group, AMX in the microbial methanogenesis system degraded rapidly, with 54.2% degraded after 2 days and 83.8% after 10 days, reducing its concentration to 0.16 × 10⁵ u/L. By contrast, the control group only degraded 28.3% over the same period. These results indicate that the microbial methanogenesis system effectively degrades AMX, thereby minimizing its environmental impact.

To examine changes in ARGs 30 days after the addition of antibiotics, the distribution of ARGs analyzed is presented in Fig. 8b. It can be observed that the main types of ARGs did not change significantly before and after the addition of antibiotics. ARGs with relative percentages exceeding 20% include glycopeptide and tetracycline, while the proportion of ARGs attributed to β-lactam antibiotics before and after addition were 2.97% and 3.61%, respectively. Figure 8c shows the changes in ARGs before and after the addition of antibiotics. ARGs that decreased include glycopeptide (0.50%), aminoglycoside (0.76%), and phenicol (0.17%). ARGs that increased over time include tetracycline (1.16%), beta-lactam (0.64%), cephalosporin (0.11%), and sulfonamide (0.08%). These findings indicate that after prolonged cultivation with the addition of β-lactam antibiotics, the distribution of ARGs remains largely consistent with that before the addition, with the most significant increase in β-lactam ARGs being only 0.64%. Other resistance genes did not exhibit significant growth. However, the microbial methanogenesis system has a weaker ability to remove ARGs compared to anaerobic digestion, likely due to the absence of hydrolysis and acidogenesis stages in this system, missing the hydrolysis and acidogenesis stages where bacteria related to the removal of resistance genes are present. In summary, β-lactam antibiotics do not lead to a substantial increase in ARGs during the methanogenesis process. It should be clarified that the effluent from this system still requires additional methods to effectively remove residual antibiotics before discharge.

To advance the practical application of this research, future studies should prioritize the following aspects: First, pilot-scale experiments are essential to assess the system’s feasibility under the antibiotic influence in real-world conditions, focusing on maintaining methane production efficiency during scaling and exploring optimized designs, such as modular configurations or advanced mixing techniques. Second, a comprehensive economic feasibility analysis should be performed, comparing operational costs with potential revenues and further reducing expenses by utilizing low-cost substrates, such as flue gas CO₂. Third, efforts should be directed toward optimizing system performance by adjusting operational parameters or supplementing nutrients to enhance microbial activity and carbon fixation efficiency. Moreover, to ensure environmental compliance, effective techniques for residual antibiotic removal, such as membrane filtration or ozonation, should be integrated to meet discharge standards. Collectively, these measures will provide a robust scientific foundation for the practical implementation of this research while addressing potential challenges in real-world applications.

### Conclusion

This study examines the selective evolution effect of β-lactam antibiotics in enhancing methane production. The findings reveal that β-lactam antibiotics, exemplified by AMX, improved methane yield and the carbon conversion rate from substrates by 3.89 times and 15.5%, respectively. The principal mechanism of action of AMX is by inhibiting the DAla-DAla transpeptidase and carboxypeptidase activity of the PBPs required for peptidoglycan biosynthesis, selectively inhibiting interfering bacteria such as *Sulfurospirillum* and *Desulfovibrio* (with inhibition rates of 50.6% and 49.6%, respectively). By contrast, AMX does not inhibit methanogens; instead, the inhibition of interfering bacteria facilitates a 61.51% increase in methanogen growth. Meanwhile, AMX is rapidly degraded, with ARGs increasing by only 0.64%, indicating smaller environmental side effects. The use of carbon sources directly utilizable by methanogens, such as sodium acetate and CO₂, in combination with the selective evolution effect of β-lactam antibiotics, addresses the challenges posed by multiple synthesis steps and interfering microbial species in methanogenesis. This approach enhances process controllability and methane production efficiency. Furthermore, the strategy of inhibiting interfering bacteria offers valuable insights for industries related to microbial fermentation and catalytic conversion.

## Data Availability

The sequencing data have been uploaded to NCBI, BioProject PRJNA1223818. The relevant supplement materials have been uploaded to Figshare.
